# The Onerous task of managing paroxysmal nocturnal hemoglobinuria in a Low resource setting: a case report. A hematologist's experience

**DOI:** 10.4314/ahs.v24i3.53

**Published:** 2024-09

**Authors:** Musa Kasadhakawo Waiswa, Christine Sekaggya-Wiltshire, Emmanuel Seremba, Henry Ddungu, Richard Mutyabule, David Enoch Kawalya, Madeleine Verhovsek

**Affiliations:** 1 Department of Medicine, Makerere University College of Health Sciences; 2 Uganda Cancer Institute; 3 Brigham and Women's Hospital Boston MA; 4 Lancet laboratories, Uganda; 5 McMaster University, Hamilton, Canada

**Keywords:** Thrombosis, hemolysis, complement, paroxysmal nocturia, hemoglobinuria

## Abstract

**Introduction:**

Paroxysmal nocturnal hemoglobinuria (PNH) is a form of red cell membrane defect characterized by increased sensitivity to complement-mediated cell lysis, resulting in intravascular hemolytic anemia, passage of hemoglobin-containing urine, a high risk of venous thrombosis and progression to pancytopenia.

The diagnosis of PNH is based on the flow cytometric (FCM) detection of peripheral blood cell clones. Such clones lack expression of the surface molecules linked to the glycosylphosphatidylinositol (GPI) anchors. The underlying defect is a somatic mutation of the short arm of the phosphatidylinositol glycan class A gene (PIG-A).

**Case report:**

We report a case of a 34-year-old male, with recurrent hemolytic anemia and dural venous sinus thrombosis found to have PNH by flow cytometry. He is currently on anticoagulation, iron and folate supplements, intermittent steroids for hemolytic episodes as we await complement inhibitors.

**Conclusion:**

As part of the diagnostic workup for patients presenting with recurrent hemolytic anemia and thrombosis in unusual sites, clinicians should include PNH on the list of differential diagnoses. Effort should be taken to characterize the red urine reported on the urine dipstick as blood/hemoglobin by microscopy to differentiate hematuria and hemoglobinuria and order for flow cytometry as this has implications on patient management.

## Case report

**Figure 1:**
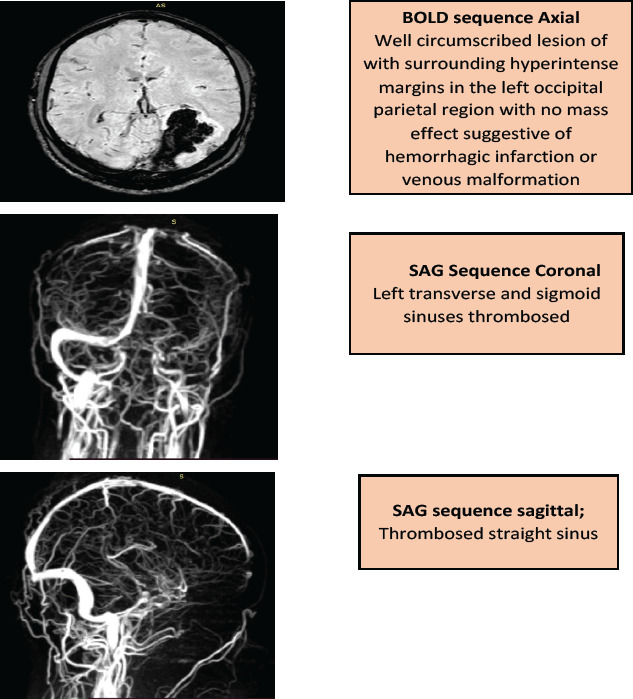
Magnetic Resonance Angiography findings

**Figure 2:**
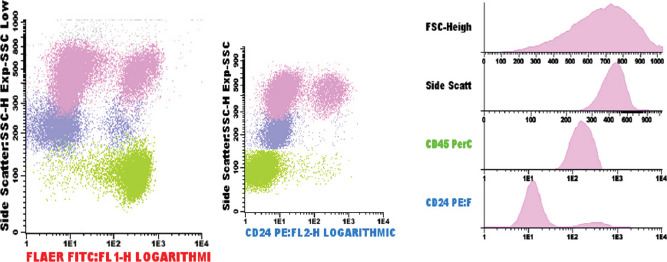
As shown in Fig.2, 81.1% of neutrophils lacked expression of GPI-linked markers FLAER and CD 24 ( Pink dots)

**Figure 3:**
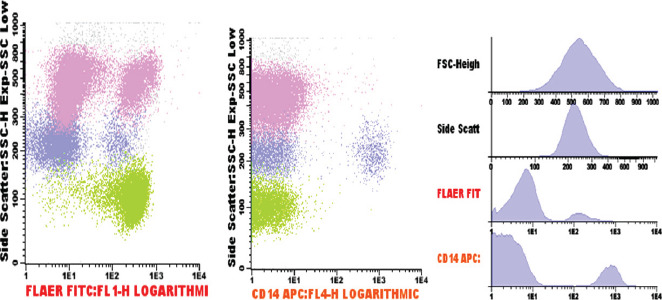
As shown in Fig.3, 88% of monocytes lacked expression of CD14 and FLAER ( purple dots)

**Figure 4:**
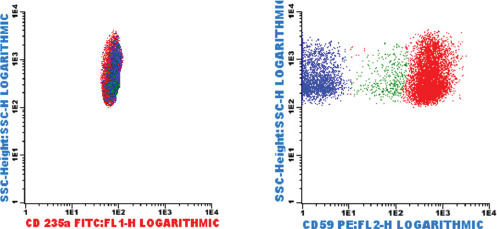
As shown in Fig.4, 25.6% of red cells had reduced or lacked expression of CD59; 22.1% were type III cells( blue dots) 3.5% were type II cells( green dots)

Between October 2018 and January 2019, a 34-year-old male was unwell, and hospitalized for the management of symptomatic anemia (hemoglobin ranging from 5.9-10.9 g/dL), “hematuria” (Blood/hemoglobin (HB): ++++, protein: ++) and a febrile illness for which empirical antibiotics were instituted and transfusion support given. He also developed acute kidney injury (creatinine 342µmol/L, urea 5.4mmol/L) which resolved on conservative management. Because of severe headache and visual impairment, he underwent a magnetic resonance angiography which revealed dural venous sinus thrombosis-stage 4 involving straight sinus, left transverse and sigmoid sinuses with hemorrhagic infarction in the left parietal, left occipital lobe, and the medial occipital temporal gyrus [Fig.1]. Anticoagulation was started but later stopped due to persistent “hematuria”. Despite this treatment, the patient did not improve and hematologists were consulted. Flow cytometry revealed the following: 81.1% of the neutrophils lacked expression of GPI-linked markers (Fluorescently Labelled Aerolysin (FLAER), and CD24 [Fig.2 Pink Dots]; 88.0% of the monocytes lacked expression of CD14 and FLAER [Fig.3 Purple Dots]; 25.6% of the red cells had reduced or lacked expression of CD59 marker; 22.1% were type III cells [complete deficiency Fig.4 Blue Dots] and 3.5% were type II cells [partial deficiency Fig.4 Green Dots].

Immunological parameters are summarized in Table 1. Bone marrow aspirate and biopsy analysis neither showed evidence of hypoplastic marrow nor myeloproliferative neoplasm. All these findings were consistent with classical PNH. The patient is currently stable on thromboprophylaxis using warfarin, iron supplements and steroid therapy during disease flares.

**Table 1:** Immunological parameters

Marker	Population gated	Results (%)	Comments
CD55	Singlets (all cells)	Non-specific binding	81.1% of the neutrophils lack expression of GPI-linked markers (FLAER, and CD24).
CD59	Singlets (all cells)	Non-specific binding	
CD59RBC	RBCs	78.5	88.0% of the monocytes lack expression of CD14 and FLAER.
CD64	Monocytes	100.0	
CD14	Monocytes	9.7	25.6% of the red cells have reduced or lack expression of CD59.
FLAER	Neutrophils	18.9	
CD24	Neutrophils	18.9	22.1% are type III cells (complete deficiency) and
			3.5% are type II cells (partial deficiency)

## Discussion

Paroxysmal nocturnal hemoglobinuria (PNH) is characterized by complement-mediated chronic hemolytic anemia, acquired thrombophilia, and bone marrow failure. The disease arises from a somatic mutation in the phosphatidyl-inositol glycan class A gene (PIG-A), disrupting glycosylphosphatidylinositol (GPI) synthesis. This molecule anchors several proteins to the cell membrane. The mutation results in the reduction or complete absence of the GPI-anchored cell surface proteins such as the complement-inhibiting proteins, CD55 (DAF-decay accelerating factor) and CD59 (MIRL-membrane inhibitor of reactive lysis), which renders the cells susceptible to complement-mediated destruction resulting into intravascular hemolysis and anemia^1^. The absence of these Cluster of Differentiation (CD) markers on PNH cells is confirmed by flow cytometry. PNH has been classified into three main categories; 1. Classical PNH, with clinical evidence of intravascular hemolysis but no bone marrow abnormalities, 2. PNH in the context of other primary bone marrow disorders such as aplastic anemia or myelodysplastic syndrome with cytogenetic abnormalities, and 3. Subclinical PNH, in which patients have small PNH clones but no clinical or laboratory evidence of hemolysis^2^. Our patient met the criteria for classical PNH with intravascular hemolysis and no bone marrow abnormalities. PNH has been described as the most vicious acquired thrombophilic state known in medicine, underscoring the high risk of thrombosis and associated morbidity and mortality in untreated patients^3^. The mechanism of thrombosis in PNH is multifactorial, involving complex interactions between the coagulation and complement cascades. Complement activation contributes to the prothrombotic tendency of PNH patients. Complement component, C5a leads to proinflammatory and prothrombotic processes by generating inflammatory cytokines such as interleukin-6, interleukin-8, and tumour necrosis factor-α. Coagulation factors can also in turn activate the complement cascade creating a vicious cycle^4^,^5^.

Based on recent clinical and laboratory evidence, it is now clear that complement is the primary driver of thrombosis in PNH^6^,^7^. Other mechanisms of thrombosis include; high levels of free hemoglobin leading to scavenging of nitric oxide (NO), which leads to platelet activation and aggregation, defective fibrinolysis from the absence of GPI-linked proteins such as urokinase-type plasminogen activator receptor and heparan sulphate and endothelial damage^7^. In a clinical trial by Hillmen et al. 2007 on the effect of the complement inhibitor eculizumab on thromboembolism, most thrombotic events were venous in nature (85%)^8^. Common sites for thrombosis include: intraabdominal with the most frequent sites being hepatic, portal, mesenteric and splenic veins, and cerebral, including the sagittal and cavernous veins^9^,^10^. Thrombosis may occur in any PNH patient, but those with a large percentage of PNH cells (>50% granulocyte clone) are at a higher risk^11^. This patient presented with thrombosis in an unusual site, dural venous sinus, and was found to have a PNH granulocyte clone of 80%, predisposing him to a high risk of thrombosis.

The main clinical manifestations of PNH include anaemia, dysphagia, abdominal pains, erectile dysfunction, renal impairement, thrombosis and pulmonary hypertension among others. The anaemia results from red cell destruction and lack of production due to underlying bone marrow failure or iron deficiency. Dysphagia, abdominal pain and erectile dysfunction result from the depletion of nitric oxide due to free haemoglobin in circulation, causing increased smooth muscle tone. Renal failure is a result of direct heme-toxicity due to hemoglobinuria, renal iron deposition or microtubular thrombosis causing renal tubular damage. Hemoglobinuria among PNH patients in international registries has been reported to be as high as 45% and 60%^12^,^13^. Pulmonary hypertension is a consequence of nitric oxide depletion in the pulmonary circulation and pulmonary embolization. In a retrospective analysis of 301 patients enrolled in the Korean National PNH Registry, the incidence of thrombosis was 18% in the pre-eculizumab era. Thromboembolism, renal impairment, and PNH-cytopenia were independent risk factors for mortality at 14-fold,8-fold and 6.2-fold respectively greater than that of age and sex matched general population^14^,^15^. The diagnosis of PNH is by flow cytometry^16^, and the standard of care involves the use of complement C5 inhibitors.

### Management of patients with PNH

Management of PNH involves simultaneous interventions against the severe hemolytic anemia, thrombosis, cytopenias, and other complications. Management of PNH depends on the classification system discussed earlier. Subclinical PNH does not usually require specific PNH therapy, but attention should be put on marrow failure syndrome. Patients with classical PNH have been shown to benefit from complement inhibitors such as monoclonal antibodies like eculizumab, small molecule inhibitors such as danicopan, cyclic peptides like pegcetacoplan, small interfering RNA (siRNA) among others. Patients with PNH/Bone Marrow Failure (BMF) with significant PNH clones may also benefit from eculizumab^17^,^18^.

### Complement Blockade by anti-C5 monoclonal antibodies

Before the monoclonal antibody, eculizumab was available, 29-43% of patients with PNH were reported to have at least one thrombosis event during their disease^19^-^21^. The 5-year survival rate for PNH patients in the pre-eculizumab period was 66.8%, which is significantly worse than the 5-year survival rate for PNH patients treated with eculizumab of 95.5%.

**Figure 5:**
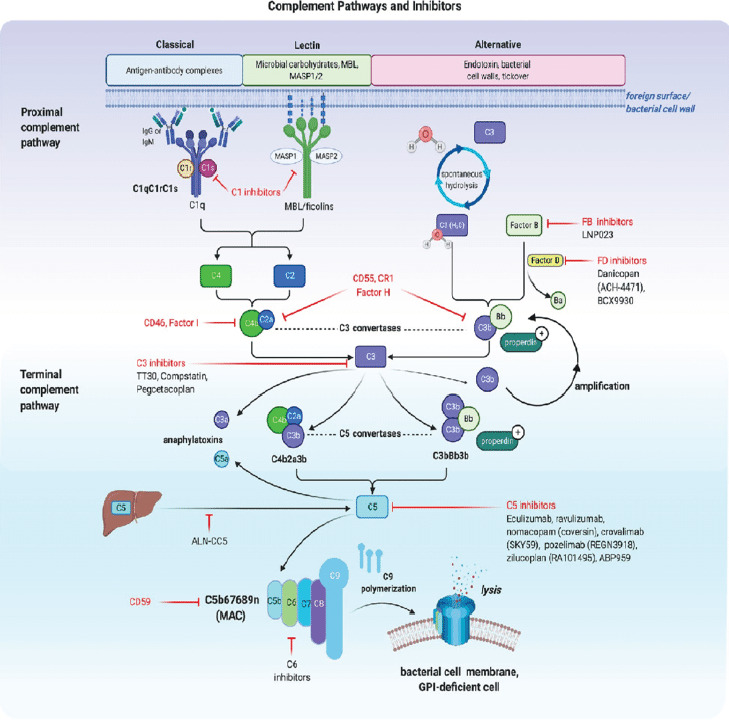
Fig.5 illustrates therapeutic targets for approved and trial complement inhibitors and future complement inhibition targets for managing complement driven disease conditions like PNH. Figure created by Biorender^36^

In 2007, a new era began for the comprehensive management of patients with PNH following the development of terminal complement inhibitors [Fig.5]. Terminal complement inhibition increases the risk of neisseria infection thus all patients should be vaccinated against Neisseria and also take appropriate prophylactic antibiotics. In a double-blind, randomized, placebo-controlled trial by Hillmen et al., eculizumab, United States FDA approved medication for PNH reduced intravascular hemolysis by 85.8%, resulted in transfusion independence and improvement in patients quality of life. Eculizumab was also associated with an absolute reduction of 6.3 thromboembolic events per 100 patient-years^5^,^8^. Challenges with this treatment include; breakthrough hemolysis, refractory states or sub-optimal responses, the impact of bimonthly intravenous therapy on work and social life, and high cost. Such challenges gave birth to ravulizumab, another FDA-approved antibody, as a complementary option for PNH patients [Fig.5]. Ravulizumab was shown to be non-inferior to eculizumab for treating PNH in patients naïve to complement inhibitor therapy and those who previously received standard-dose eculizumab. With a more convenient dosing schedule of once every eight weeks, fewer episodes of breakthrough hemolysis through a more reliable C5 blockade, and lower cost, ravulizumab became an attractive option for patients with PNH who qualify for complement inhibition therapy^22^-^24^.

### Next generation Complement inhibitors for PNH patients

Eculizumab and ravulizumab prevent intravascular hemolysis and thrombosis among PNH patients however, more than 50% of patients with PNH on C5 inhibitors have mild to moderate symptoms from PNH, and up to 20% still need occasional transfusions. Extravascular hemolysis remains a common reason for the persistent anemia. Other C5 inhibitors such as crovalimab and pozelimab have shown good efficacy in achieving disease control and are well tolerated thus providing new treatment alternatives for PNH patients once fully approved. A C3 complement component inhibitor [Fig.5], pegcetacoplan is FDA approved for treating adult patients with PNH and can block intravascular and extra-vascular hemolysis. Pegcetacoplan was compared head-to-head with eculizumab in a phase III clinical trial and found to be superior to eculizumab in improving clinical and hematological outcomes in patients with PNH25. In randomized phase III multi-centre trial assessing the efficacy and safety of pegcetacoplan in complement-inhibitor naïve patients, pegcetacoplan was superior to the control group (supportive care) in hemoglobin stabilization in 85% of patients compared to 0% in the control group. Furthermore, there were no cases of thrombosis or meningitis in the pegcetacoplan arm^26^. Small molecules selectively inhibiting either proximal alternative pathway complement factor D such as danicopan, BCX 9930, and vemircopan or factor B inhibitors are gradually gaining access to the PNH treatment space. They have shown improvements in baseline hemoglobin both as add-on treatments to the standard of care C5 Inhibitors and as well as monotherapies. Unlike the standard of care medications, these small molecule inhibitors have the advantage of being taken orally^27^-^30^. In a phase III APPOINT-PNH trial on efficacy and safety of iptacopan monotherapy in adult PNH patients naïve to complement inhibitor therapy, iptacopan increased hemoglobin levels by 2g/dL or more compared with the baseline in 92% of patients in the abscence of red cell transfusions at 24 weeks. Upto 63% of patients achieved hemoglobin levels of atleast 12g/dL without the need for transfusion^31^. In a related phase III, AP-PLY-PNH study, iptacopan increased baseline hemoglobin by 2g/dL or more in 82% of complement C5 inhibitor experienced iptacopan patients compared to 0% in patients who did not use iptacopan at 24 weeks in the absence of red cell transfusion^32^. These trials led to its recent approval by the United States FDA as the first oral monotherapy for treating adult patients with PNH. As shown in Fig.5 there are a number of other potential therapeutic targets for complement inhibition, some of which may have a role in treating PNH patients such as C1 inhibitors, C6 Inhibitors, CD46 agonists. CD46 is a non GPI-linked complement inhibitor on nucleated cells and probably the reason why there is no evidence of reduced leukocyte life span in PNH patients. This is because of its anti-complement properties and therefore, CD46 agonists are potential therapeutic targets in PNH patients. This is, therefore another fertile ground for future therapeutic research.

Severe hemolytic anemia from intravascular hemolysis involves transfusion support with red cells, folic acid 5mg daily, iron supplementation titrated against serum transferrin saturation levels, and steroid therapy. A short course intermittent low-dose prednisone has been used to control hemolysis, borrowing from its effectiveness in treating autoimmune hemolysis. There is however no evidence that prednisone inhibits complement-mediated hemolysis rather, it can prevent acute inflammation-associated triggers of PNH hemolysis. Our patient often receives short course low dose steroids during hemolytic episodes cognizant of the drug-related toxicities. He also receives iron and folate supplements.

### Thrombosis in PNH

Use of anticoagulants like heparin or low-molecular-weight heparins, then bridging to vitamin K antagonist therapy are the first actions to take in the setting of acute thrombotic events in patients with PNH. Anticoagulation is also recommended in patients without eculizumab but with high PNH clones (granulocyte clone >50%), pregnancy, high level of D-dimer, and other thrombophilic states. Primary prophylaxis with warfarin in PNH prevents thrombosis^3^, ^11^, ^17^, ^22^. Our patient was initiated on anticoagulation following confirmation of PNH clones and continues on dose adjusted warfarin to the present date, fortunately without any breakthrough thrombotic events. Novel anticoagulants can be used in treating thrombosis associated with PNH, however, data is still limited and are primarily reserved for low-risk PNH patients. Their efficacy in high-risk subgroups, including those with past thromboembolism, active hemolysis, or large clone PNH is however less clear^33^,^34^. Regardless of the type of anticoagulants used, patients with PNH must be monitored as they carry a life-long risk of thrombosis and re-thrombosis unless complement inhibition is initiated.

### Clinical course to date

In the initial months of the diagnosis, there was sub-optimal anticoagulation due to frequent withholding of medication each time the patient presented with “hematuria”. Despite sub-optimal prothrombin times from different laboratories, this was erroneously thought to be hematuria following supratherapeutic dosing. On further evaluation however, -in particular the absence of red blood cells on urine microscopy, it was determined that the “hematuria” was actually hemoglobinuria^35^, which is consistent with the presentation of PNH and does not warrant interrupting anticoagulation during the acute hemolytic episodes. He has since been on dose-adjusted vitamin K antagonist, warfarin, folic acid, iron supplements and short course steroid therapy. He seems to benefit from steroid therapy during hemolytic episodes despite absence of supportive evidence for their use in PNH patients. No evidence of steroid toxicity has been documented to date since he uses low dose steroids and only during hemolytic episodes. He is stable, has not suffered any breakthrough thrombotic event in the last four years although he continues to get between three and five hemolytic episodes a year. Our patient has not been able to access eculizumab or related medications since they are not readily available in sub-saharan Africa.

## Conclusion

PNH remains a disease with a pleiotropic clinical presentation, and its treatment should be based on the actual clinical presentation of the patient. The wide range of differential diagnoses for hemolytic anemias in a predominantly infectious diseases setting, the clinical mimicry of hematuria and hemoglobinuria requiring additional workup, optimization of anticoagulation for thrombotic events in the face of presumed bleeding (hematuria vs hemoglobinuria), the lack of access to flow cytometry which is the comfirmatory diagnostic test of choice, the lack of access and affordability of complement C5 inhibitors and related molecules which is a standard of care, the rarity of PNH cases in comparison to the infectious disease burden in the developing world and the associated prudent budgetary allocations limiting available resources for diagnostic and therapeutic options for PNH, and the rare risk of clonal evolution to MDS, acute myeloid leukaemia and/or aplastic anemia make(s) the diagnosis and management of PNH an onerous task for clinicians in the low resource settings. In low-income countries, where access to complement inhibitors is limited, a thromboprophylaxis strategy in PNH patients can treat thrombosis and possibly prevent recurrent thrombosis, a leading cause of death in PNH patients. Because of the rarity of the disease, PNH patients should be included in prospective PNH registries to offer new insights into the natural course of the disease through information exchange and access to novel therapies through research collaborations.
